# Comprehensive Atlas of Alternative Splicing Reveals NSRP1 Promoting Adipogenesis through *CCDC18*

**DOI:** 10.3390/ijms25052874

**Published:** 2024-03-01

**Authors:** Lei Liu, Wei Wang, Weiwei Liu, Xingzheng Li, Guoqiang Yi, Adeyinka Abiola Adetula, Haibo Huang, Zhonglin Tang

**Affiliations:** 1 Key Laboratory of Livestock and Poultry Multi-Omics of MARA, Agricultural Genomics Institute at Shenzhen, Chinese Academy of Agricultural Sciences, Shenzhen 518124, China; liulei03@caas.cn (L.L.); wwlucky1005@163.com (W.W.); liuww19990703@163.com (W.L.); lxz2019@alu.cau.edu.cn (X.L.); yiguoqiang@caas.cn (G.Y.); huanghaibo@caas.cn (H.H.); 2 Shenzhen Branch, Guangdong Laboratory of Lingnan Modern Agriculture, Agricultural Genomics Institute at Shenzhen, Chinese Academy of Agricultural Sciences, Shenzhen 518124, China; 3 Kunpeng Institute of Modern Agriculture at Foshan, Agricultural Genomics Institute, Chinese Academy of Agricultural Sciences, Foshan 528226, China; 4 Reproductive Biotechnology, Department of Molecular Life Sciences, TUM School of Life Sciences, Technical University Munich, 85354 Freising, Germany; quueenyk10@yahoo.com

**Keywords:** pigs, alternative splicing, NSRP1, *CCDC18*, adipose deposition

## Abstract

Alternative splicing (AS) plays a crucial role in regulating gene expression, function, and diversity. However, limited reports exist on the identification and comparison of AS in Eastern and Western pigs. Here, we analyzed 243 transcriptome data from eight tissues, integrating information on transcription factors (TFs), selection signals, splicing factors (SFs), and quantitative trait loci (QTL) to comprehensively study alternative splicing events (ASEs) in pigs. Five ASE types were identified, with Mutually Exclusive Exon (MXE) and Skipped Exon (SE) ASEs being the most prevalent. A significant portion of genes with ASEs (ASGs) showed conservation across all eight tissues (63.21–76.13% per tissue). Differentially alternative splicing genes (DASGs) and differentially expressed genes (DEGs) exhibited tissue specificity, with blood and adipose tissues having more DASGs. Functional enrichment analysis revealed coDASG_DEGs in adipose were enriched in pathways associated with adipose deposition and immune inflammation, while coDASG_DEGs in blood were enriched in pathways related to immune inflammation and metabolism. Adipose deposition in Eastern pigs might be linked to the down-regulation of immune-inflammation-related pathways and reduced insulin resistance. The TFs, selection signals, and SFs appeared to regulate ASEs. Notably, ARID4A (TF), NSRP1 (SF), *ANKRD12*, *IFT74*, *KIAA2026*, *CCDC18*, *NEXN*, *PPIG*, and *ROCK1* genes in adipose tissue showed potential regulatory effects on adipose-deposition traits. NSRP1 could promote adipogenesis by regulating alternative splicing and expression of *CCDC18*. Conducting an in-depth investigation into AS, this study has successfully identified key marker genes essential for pig genetic breeding and the enhancement of meat quality, which will play important roles in promoting the diversity of pork quality and meeting market demand.

## 1. Introduction

As an essential livestock animal, pigs provide abundant food resources for human beings and hold significant symbolic importance in various cultures. The Eastern and Western pig populations, as the main subtypes, have distinct ecological environments and developmental histories [1]. Eastern pigs are mainly distributed in Asia, including China, Japan, South Korea, and other countries, and their history of communication with humans can be traced back tens of thousands of years ago [2,3]. On the other hand, Western pigs originated in Europe and have spread to other continents through human exploration and expansion [4]. Throughout their long domestication process, these two pig types have developed distinct genetic characteristics and ecological habits [4,5]. For example, Western pigs have a faster growth rate, while Eastern pigs have stronger disease resistance and fat content [6,7,8].

The alternative splicing (AS) of pre-RNA is a crucial transcriptional regulatory mechanism involving splicing mRNA precursors of genes in different ways. This process allows a single gene to generate multiple transcripts, translating diverse proteins and significantly enriching the transcriptome and proteome diversity [9,10]. AS plays a pivotal role in organism growth, development, and cell differentiation [11] and is widespread in mammals [12]. In humans, 40–60% of genes undergo AS, enhancing the coding capacity of the genome [13]. Furthermore, AS is linked to numerous disease factors [14], underscoring its critical importance. There are many factors that influence AS, such as transcription factors, selection signals, splicing factors, etc. [15,16,17,18].

Studying AS in pigs will deepen our understanding of pig gene functionality and regulatory mechanisms and shed light on their relationships and evolutionary processes with other species. Moreover, as society progresses and concerns about food safety and environmental protection grow, pigs have garnered increased attention as a major livestock animal [19]. Investigating AS characteristics in Eastern and Western pigs can offer valuable insights into pig domestication and breeding, leading to improvements in productivity and adaptability and contributing to the sustainable development of the pork industry. Researchers have identified widespread pre-RNA AS occurrences in multiple pig tissues [20,21,22]. However, the differences in AS among various tissues of Eastern and Western pigs and their molecular regulatory mechanisms remain unclear.

In this study, we collected a total of 243 transcriptome datasets from eight tissues (adipose, blood, heart, lung, kidney, muscle, ovary, and spleen) in Eastern and Western pigs from public databases. By integrating information on transcription factors (TFs), selection signals, splicing factors (SFs), and quantitative trait loci (QTL), we comprehensively compared AS characteristics between the two pig populations. The results revealed tissue-specific differences in both differentially alternative splicing genes (DASGs) and differentially expressed genes (DEGs), potentially influenced by TFs, selection signals, and SFs. Furthermore, by incorporating QTLs information, we identified marker genes associated with meat quality traits such as backfat thickness and intermuscular fat deposition. Our research aims to provide scientific evidence for the conservation and rational utilization of Eastern and Western pig genetic resources. Additionally, we seek to establish a new theoretical foundation for pig genetic breeding and the improvement of meat quality traits, thereby contributing to the sustainable development of the livestock industry and deepening our understanding of pig gene function and regulatory mechanisms.

## 2. Results

### 2.1. Genome-Wide Identification of Alternative Splicing Events (ASEs) in Different Tissues

**Figure 1:**
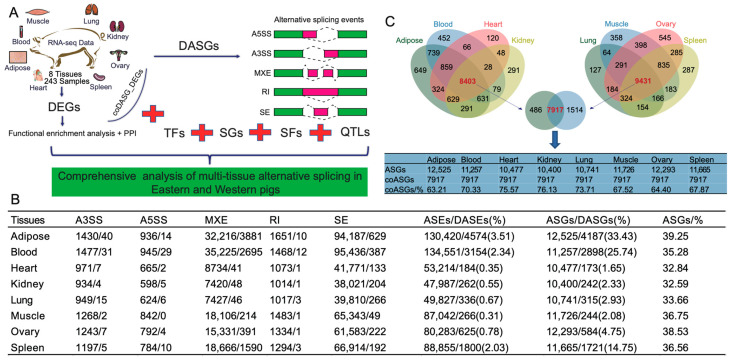
Identification of alternative splicing across tissues. (**A**) Experimental design and flowchart. DEGs = Differentially Expressed Genes; DASGs = Differentially Alternative Splicing Genes; coDASG_DEGs = Common genes between DASGs and DEGs; PPI = Protein-Protein Interaction; A5SS = Alternative 5′ Splice Site; A3SS = Alternative 3′ Splice Site; MXE = Mutually Exclusive Exon; SE = Skipped Exon; RI = Retained Intron; TFs = Transcription Factors; SGs = Genes under Selection; SFs = Splicing Factors; QTLs = Quantitative Trait Loci. (**B**) Alternative splicing events and genes in multiple tissues. ASEs = Alternative Splicing Events; DASEs = Differentially Alternative Splicing Events; ASGs = Alternative Splicing Genes; DASGs = Differentially Alternative Splicing Genes; ASGs/% = The proportion of ASGs in all genes of the pig genome; the values before and after the slash of A3SS, A5SS, MXE, RI and SE represent the number of ASEs and DASEs, respectively; the numbers in parentheses of DASEs/ASEs(%) indicate the percentage of DASEs in ASEs; the numbers in parentheses of DASGs/ASGs(%) indicate the percentage of DASGs in ASGs. (**C**) Conservation of alternative splicing across tissues. The panel above indicates the overlapped ASGs among different tissues. The bottom panel represents statistics on the number of ASGs in different tissues. coASGs = Common ASGs among the eight tissues.

To investigate the differences in alternative splicing events (ASEs) across various tissues of Eastern and Western pigs, we conducted identification and differential analysis of five types of ASEs in the eight tissues, including Alternative 3′ Splice Site (A3SS), Alternative 5′ Splice Site (A5SS), Mutually Exclusive Exon (MXE), Retained intron (RI), and Skipped Exon (SE) (Figure 1A). The results revealed that MXE and SE types of ASEs and differentially alternative splicing events (DASEs) had the highest occurrence in each tissue. Among them, SE ASEs were the most abundant, while MXE had the highest number of DASEs (Figure 1B). In terms of different tissues, blood and adipose tissues exhibited a higher number of ASEs and DASEs. Additionally, these two tissues had the highest proportion of DASEs, with adipose showing 1.5 times (3.51%/2.34%) more than that of blood (Figure 1B). We further counted the number of genes with alternative splicing (ASGs) and found that the percentage of genes undergoing AS in each tissue ranged from 32.59% to 39.25% of the genome-wide genes (Figure 1B), indicating that the number of genes undergoing AS in each tissue was relatively stable. Moreover, we observed that the proportion of differentially alternative splicing genes (DASGs) was highest in adipose and blood tissues (33.43% and 25.74%, respectively), with adipose showing 1.3 times more than blood (Figure 1B). This finding suggested that AS plays a significant role in adipose deposition of pigs. Furthermore, we investigated conserved genes with AS (ASGs) in different tissues and identified 7917 common ASGs present in all eight tissues, accounting for 63.21% to 76.13% of ASGs in each tissue (Figure 1C). This finding suggested that genes undergoing AS in various tissues display a certain level of conservation.

### 2.2. Tissue-Specific Differentially Alternative Splicing Genes (DASGs) and Differentially Expressed Genes (DEGs)

We further investigated the tissue-specificity of differentially alternative splicing genes (DASGs) and differentially expressed genes (DEGs) in each tissue. The results revealed that, in the comparison between Western and Eastern pigs, both DASGs and DEGs exhibited tissue-specificity, with adipose and blood tissues showing higher tissue-specificity for both categories. Among all tissues, the ovary had the highest tissue-specificity for DEGs (65.28%) but a relatively lower tissue-specificity for DASGs (17.29%), suggesting that gene expression in the ovary might be less influenced by AS (Table 1). Furthermore, we calculated the ratio of DASGs to DEGs in each tissue and found that, on average, each gene underwent 4.98, 3.02, 0.97, 1.11, 0.88, 0.79, 0.43, and 2.78 ASEs in adipose, blood, heart, kidney, lung, muscle, ovary, and spleen tissues, respectively. These results further indicated that genes in adipose and blood tissues were more likely to undergo AS compared to other tissues (Table 1), and we focused on these two tissues in the following research. We identified 840 and 960 DEGs for adipose and blood, respectively, and filtered down to 83 and 164 common genes between DASGs and DEGs (coDASG_DEGs) for adipose and blood, respectively (Table 1). These coDASG_DEGs may be significantly influenced by ASEs and represent promising candidates for further investigation.

### 2.3. Functional Analysis of DEGs Association with ASEs

**Figure 2:**
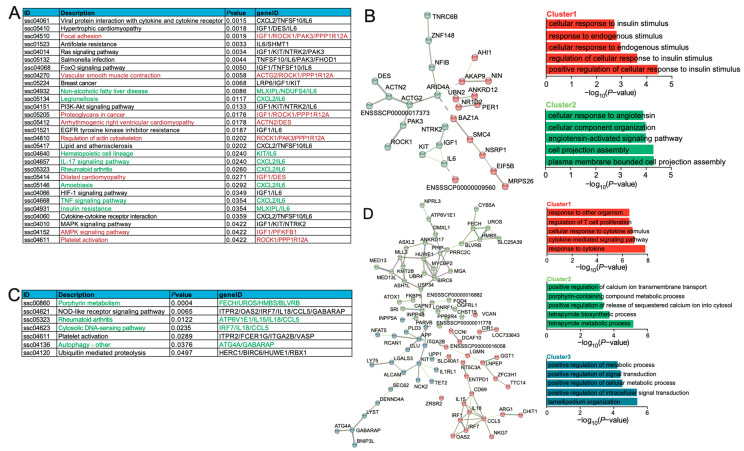
Functional enrichment analysis of the coDASG_DEGs. (**A**) Significant KEGG pathway of coDASG_DEGs in adipose (*p* < 0.05). (**B**) PPI analysis of coDASG_DEGs in adipose tissue. (**C**) Significant KEGG pathway of coDASG_DEGs in blood (*p* < 0.05). (**D**) PPI analysis of coDASG_DEGs in blood. The left panel represents the PPI predicted with the coDASG_DEGs. These genes are divided and colored into different clusters according to K-means. The right panel shows the biological process terms predicted with the genes in different clusters. The color of the cluster on the right panel is the same as the color of the gene on the left panel.

To investigate the impact of alternative splicing events (ASEs) on the biological functions in adipose and blood tissues, we performed Kyoto Encyclopedia of Genes and Genomes (KEGG) pathway enrichment analysis on coDASG_DEGs specific to these two tissues. In adipose tissue, we identified a total of 29 significantly enriched pathways (*p* < 0.05). Among them, the Ras signaling pathway, FoxO signaling pathway, PI3K-Akt signaling pathway, insulin resistance, MAPK signaling pathway, and AMPK signaling pathway were related to adipose deposition, involving genes such as *IGF1*, *MLXIPL*, *NTRK2*, *IL6*, *KIT*, *TNFSF10*, *PFKFB1*, and *PAK3* (Figure 2A). Notably, genes *IGF1* and *PFKFB1* in the AMPK signaling pathway were significantly upregulated in Eastern pigs, while genes *MLXIPL* and *IL6* in the insulin resistance pathway were significantly downregulated (Figure 2A). Furthermore, we observed that genes related to immune inflammation pathways, such as Non-alcoholic fatty liver disease, Legionellosis, IL-17 signaling pathway, Rheumatoid arthritis, Amoebiasis, and TNF signaling pathway, were significantly downregulated, involving *MLXIPL*, *CXCL2*, *KIT*, *NDUFS4*, and *IL6* (Figure 2A). Moreover, we conducted a Protein–Protein Interaction (PPI) analysis on coDASG_DEGs using the STRING database. We applied K-means clustering and divided the adipose tissue into two clusters. Cluster 1 in adipose tissue was enriched in insulin stimulus-related pathways (Figure 2B), further suggesting a possible association between adipose deposition in Eastern pigs and insulin resistance.

In blood, we identified a total of seven significantly enriched KEGG pathways (*p* < 0.05), mainly related to immune inflammation and metabolism. All coDASG_DEGs in pathways such as Porphyrin metabolism, Rheumatoid arthritis, Cytosolic DNA-sensing pathway, and Autophagy—other showed significant downregulation in Eastern pigs involving *ATG4A*, *ATP6V1E1*, *BLVRB*, *CCL5*, *FECH*, *GABARAP*, *HMBS*, *IL15*, *IL18*, *IRF7*, and *UROS* (Figure 2C). Similarly, we conducted PPI analysis on coDASG_DEGs using the STRING database for blood tissue. We applied K-means clustering and divided the blood tissue into three clusters. Cluster 1 was enriched in immune inflammation-related pathways, while Cluster 2 and Cluster 3 were primarily enriched in metabolism-related pathways (Figure 2D). These pathways and genes suggested differences in disease resistance and metabolic levels between Eastern and Western pigs.

### 2.4. Effects of Transcription Factors (TFs) on Alternative Splicing (AS)

**Figure 3:**
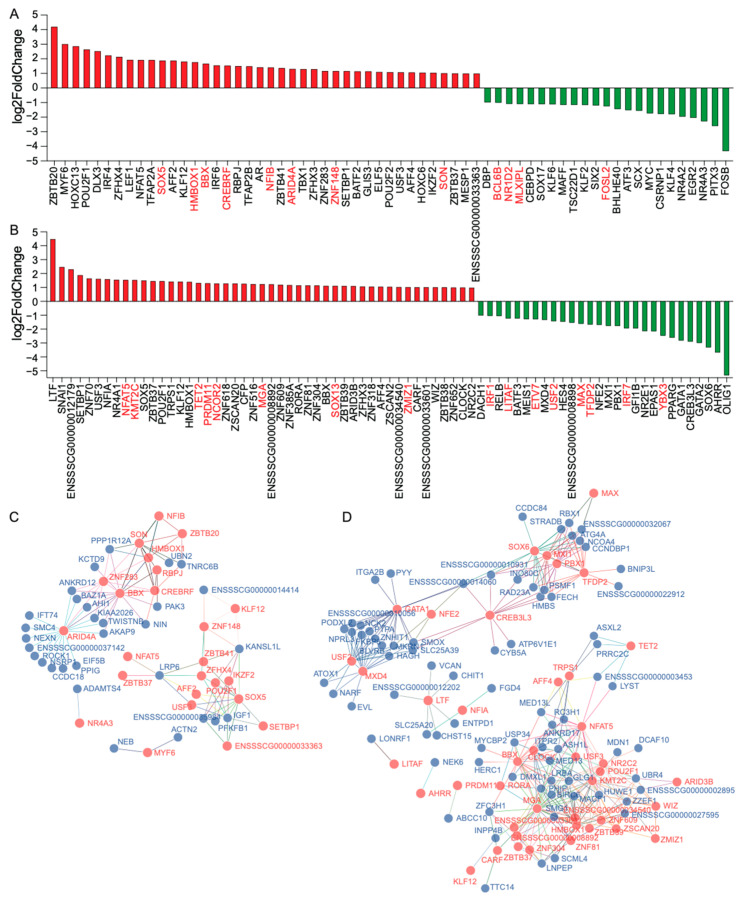
Combined analysis of the coDASG_DEGs and TFs. (**A**,**B**) TFs in DEGs of adipose (**A**) and blood (**B**). The red and green bars denote the up-regulation and down-regulation of TF in the Eastern pig, respectively. The red font indicates that the differentially expressed TF also undergoes differentially alternative splicing. (**C**,**D**) The interaction network of TF and the coDASG_DEGs in adipose (**C**) and blood (**D**). TFs and coDASG_DEGs with a correlation coefficient (r) greater than 0.9 and a *p* value less than 3.40 × 10^−14^ were screened and made into an interaction network. The red and blue dots separately represent TF and genes.

Researchers show that transcription factors (TFs) can regulate the splicing process of genes [15,16]. To investigate the regulatory role of TFs on gene AS, we conducted a joint analysis of DEGs, DASGs, and TFs. We identified 63 and 75 TFs in the DEGs (coDEG_TFs) of adipose and blood, respectively (Figure 3A,B), accounting for 7.50% and 7.81% of adipose and blood DEGs. Among them, in adipose tissue, 12 coDEG_TFs also exhibited differentially alternative splicing between Eastern and Western pigs, including *ARID4A*, *BBX*, *BCL6B*, *CREBRF*, *FOSL2*, *HMBOX1*, *MLXIPL*, *NFIB*, *NR1D2*, *SON*, *SOX5*, and *ZNF148* (Figure 3A). In terms of blood tissue, 16 coDEG_TFs also showed differentially alternative splicing between Eastern and Western pigs, including *ETV7*, *IRF1*, *IRF7*, *KMT2C*, *LITAF*, *MAX*, *MGA*, *NCOR2*, *NFAT5*, *PRDM11*, *SOX13*, *TET2*, *TFDP2*, *USF2*, *YBX3*, and *ZMIZ1* (Figure 3A).

We conducted Pearson correlation analysis to study the regulatory effect of these coDEG_TFs on coDASG_DEGs gene expression. Using the criteria *r* > 0.9 and *p* < 3.40 × 10^−14^, we identified 110 TF-gene pairs involving 24 coDEG_TFs and 38 (45.78%) coDASG_DEGs in adipose tissue (Figure 3C). In blood tissue, we found 350 TF-gene pairs involving 46 coDEG_TFs and 86 (52.44%) coDASG_DEGs (Figure 3D). These results indicate that approximately half of the coDASG_DEGs are significantly influenced by TFs, revealing the important regulatory role of TFs in gene AS.

### 2.5. The Impact of Genes under Potential Selection on Alternative Splicing

**Figure 4:**
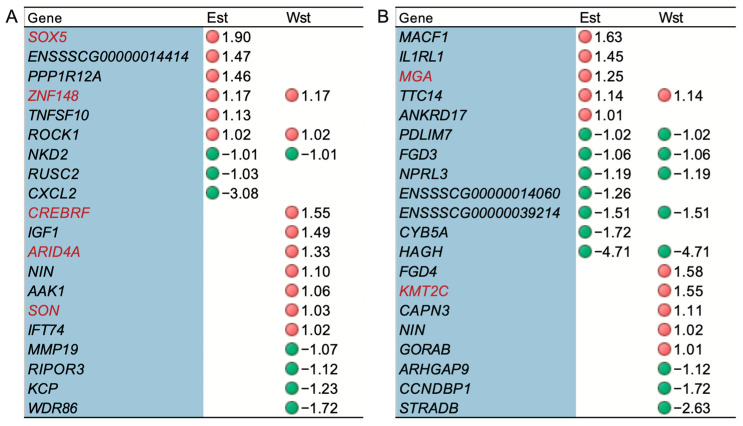
Genes under selection (SGs) in coDASG_DEGs of adipose and blood tissues. (**A**,**B**) The overlapped genes of SGs and coDASG_DEGs in adipose (**A**) and blood (**B**). Est and Wst represent SGs in Eastern and Western pigs, respectively. Red dots with positive values and green dots with negative values indicate the genes are up-regulated and down-regulated in Eastern pigs, respectively. The genes in red font are TFs.

The differences in ASEs between Eastern and Western pigs may be influenced by long-term artificial selection during domestication and breeding. Therefore, we also investigated the impact of selection signals on AS. Based on the previously identified genes under selection (SGs) in Eastern and Western pigs [6], we further explored their influence on AS. In both adipose and blood tissues, we identified 20 genes under selection intersecting with coDASG_DEGs (Figure 4). Among these, five TFs (SOX5, ZNF148, CREBRF, ARID4A, SON) were involved in adipose tissue (Figure 4A), and two TFs (MGA, KMT2C) were involved in blood tissue (Figure 4B).

In adipose tissue, we found that *SOX5*, *ENSSSCG00000014414*, *PPP1R12A*, *ZNF148*, *TNFSF10*, *ROCK1*, *NKD2*, *RUSC2*, and *CXCL2* were under selection in Eastern pigs (Figure 4A). Among them, *SOX5*, *ENSSSCG00000014414*, *PPP1R12A*, *ZNF148*, *TNFSF10*, and *ROCK1* were significantly upregulated in Eastern pig adipose tissue (Figure 4A), potentially influencing adipose deposition in pigs. In blood tissue, *MACF1*, *IL1RL1*, *MGA*, *TTC14*, *ANKRD17*, *PDLIM7*, *FGD3*, *NPRL3*, *ENSSSCG00000014060*, *ENSSSCG00000039214*, *CYB5A*, and *HAGH* were under selection in Eastern pigs (Figure 4B). Among them, *MACF1*, *IL1RL1*, *MGA*, *TTC14*, and *ANKRD17* were significantly upregulated in Eastern pig’s blood tissue (Figure 4B), potentially affecting disease resistance in pigs.

### 2.6. Effects of Splicing Factors (SFs) on Alternative Splicing

**Figure 5:**
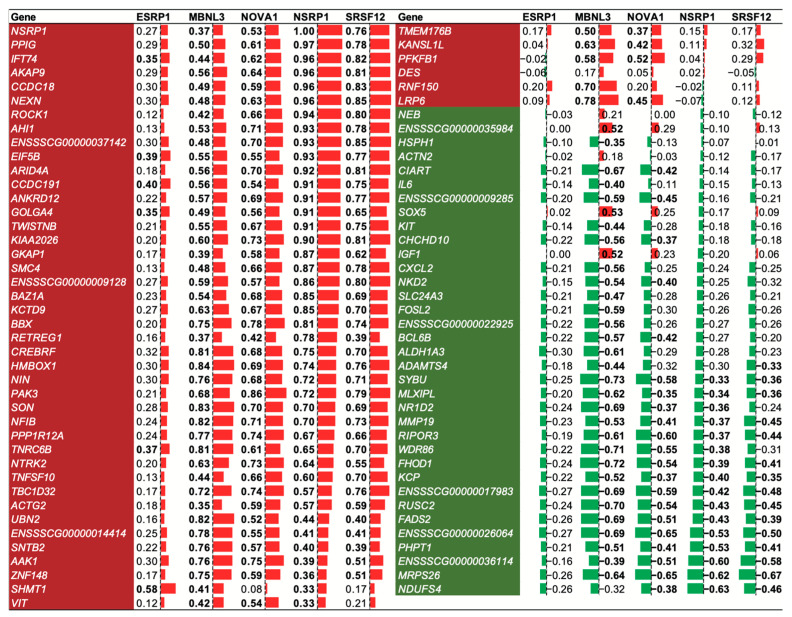
Pearson correlation analysis of SFs and coDASG_DEGs. Genes with red and green backgrounds were positively and negatively correlated with SFs, respectively. The number in the figure represents the correlation coefficient. The positive and negative values represent positive and negative correlations, accompanied by the red and green bars. The length of the bars represents the absolute value of the correlation coefficient. The number in bold font indicates a significant correlation (*p* < 0.05).

Splicing factors (SFs) are protein factors involved in the RNA precursor splicing process [17]. To study the regulatory role of SFs on alternative splicing, we identified five SFs (SERP1, MBNL3, NOVA1, NSRP1, and SRSF12) from DEGs in adipose tissue. Then, we conducted a Pearson correlation analysis between the gene expression levels of these five SFs and coDASG_DEGs. The results showed that out of 415 SF-coDASG_DEG pairs, 263 (63.37%) pairs exhibited significant correlations (|*r*| > 0.324, *p* < 0.05, Figure 5). It indicates that these five SFs play a crucial role in the alternative splicing of DEGs in the adipose tissue of Eastern and Western pigs.

Furthermore, among the 83 coDASG_DEGs, we found that 74 (89.16%) genes exhibited consistent correlation patterns with these five SFs, with 44 genes showing positive correlations and 30 genes showing negative correlations. It suggested that these SFs may play a co-regulatory role in the process of AS (Figure 5). After sorting the 415 SF-coDASG_DEG pairs in descending order of correlation coefficient, we found that the top 18 pairs of genes were significantly and highly positively correlated with the splicing factor NSRP1 (*r* > 0.86, *p* < 6.10 × 10^−12^, Figure 5). These genes included *PPIG*, *IFT74*, *AKAP9*, *CCDC18*, *NEXN*, *ROCK1*, *AHI1*, *ENSSSCG00000037142*, *EIF5B*, *ARID4A*, *CCDC191*, *ANKRD12*, *GOLGA4*, *TWISTNB*, *KIAA2026*, *GKAP1*, *SMC4*, and *ENSSSCG00000009128* (Figure 5), indicating that the SF NSRP1 may have significant regulatory effects on adipose AS and pig adipose deposition.

### 2.7. Splicing Factor NSRP1 and Correlated Genes Impact Adipose-Deposition Traits

**Figure 6:**
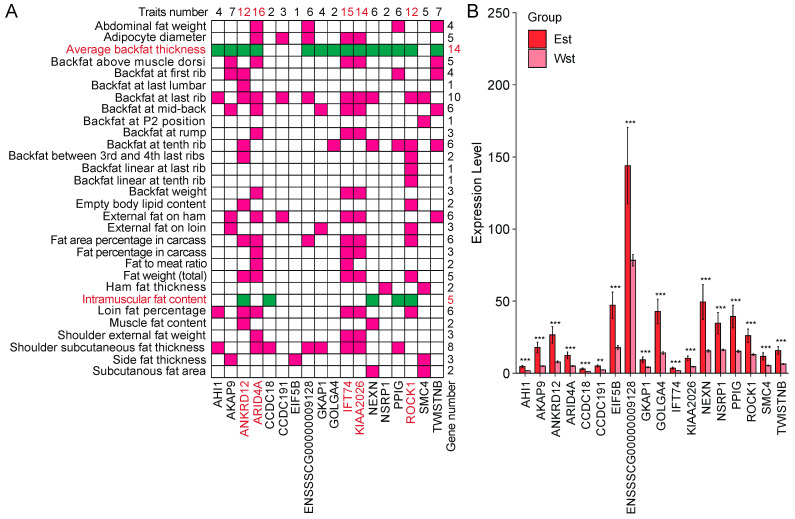
Comparison of NSRP1 and its related genes with QTLs related to adipose traits. (**A**) The information of QTLs and the related genes. The genes in the figure are splicing factor *NSRP1* and the genes highly correlated with it (*r* > 0.86, *p* < 6.10 × 10^−12^). Green and rose-red squares represent genes present in the corresponding QTLs. The numbers on the top indicate the number of QTLs in which a specific gene is present, and the numbers on the right indicate the number of genes present in a specific QTL. Genes in red font represent more than 12 QTLs related to them. (**B**) Comparison of the expression level of these genes in Eastern and Western pigs. Est: Eastern, Wst: Western. ** represents the *p*-value is less than 0.01, *** means the *p*-value is less than 0.001.

To investigate the potential role of the splicing factor NSRP1 in adipose deposition, we downloaded QTLs data for pigs from PigQTLdb (https://www.animalgenome.org/cgi-bin/QTLdb/SS/index, accessed on 23 July 2022). We then conducted a combined analysis using NSRP1 and the 18 genes highly correlated with the QTLs information. As a result, 1057 QTLs were identified, of which 235 were associated with adipose-deposition traits. These QTLs were linked to the 18 genes (excluding *ENSSSCG00000037142*) and were associated with 30 traits, including abdominal fat weight, adipocyte diameter, backfat thickness, and intramuscular fat content (Figure 6A, Table S1). Further analysis revealed that all these genes in adipose-related QTLs were highly expressed in the Eastern pigs (Figure 6B). Moreover, as many as 14 genes were found in the Average backfat thickness QTL, further indicating a potential link between *NSRP1* and its related genes with adipose deposition. Notably, five genes, namely *ANKRD12*, *ARID4A*, *IFT74*, *KIAA2026*, and *ROCK1*, appeared in 12 or more QTLs, suggesting their potentially significant roles in adipose-deposition traits (Figure 6A). Additionally, *ANKRD12*, *CCDC18*, *NEXN*, *PPIG*, and *ROCK1* were associated with the intramuscular fat content QTL (Figure 6A), implying their potential importance in intermuscular fat deposition and meat quality improvement. These findings suggested a possible regulatory network involving NSRP1 and its correlated genes that influence pigs’ adipose deposition and meat quality traits.

### 2.8. Splicing Factor NSRP1 Regulates Adipogenesis

**Figure 7:**
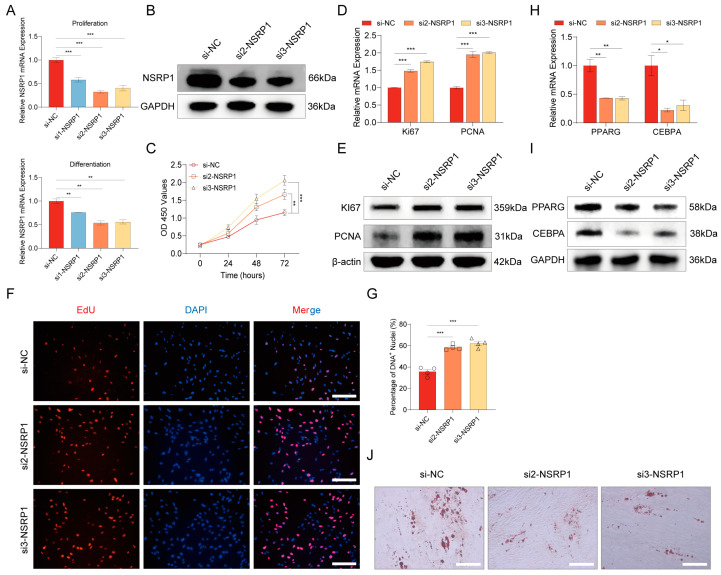
Effect of *NSRP1* on proliferation and differentiation of pre-adipocyte and lipid droplet formation. (**A**) The RT-qPCR results demonstrate the interference efficiency of three distinct small interfering RNAs targeting *NSRP1* during both cell proliferation (**top panel**) and differentiation (**bottom panel**) stages. (**B**) Western blot experiment verified the interference efficiency of si-NSRP1. (**C**) Cell proliferation rates in response to downregulated *NSRP1* expression were investigated through CCK8 assay. (**D**) The mRNA expression levels of pre-adipocyte proliferation marker genes (*Ki67* and *PCNA*) were evaluated following the downregulation of *NSRP1* expression. (**E**) The protein expression levels of pre-adipocyte proliferation marker genes (*Ki67* and *PCNA*) were evaluated following the downregulation of *NSRP1* expression. (**F**) EdU assay was carried out after si-NSRP1 transfection for 24 h. Cells undergoing DNA replication were stained by EdU (red) and cell nuclei were stained with DAPI (blue). Scale bar, 200 µm. (**G**) The percentage of DNA+ nuclei in (**F**) was quantified using ImageJ (1.52i). (**H**) The mRNA expression levels of pre-adipocyte differentiation (adipogenesis) marker genes (*PPARG* and *CEBPA*) were evaluated following the downregulation of *NSRP1* expression. (**I**) The protein expression levels of pre-adipocyte differentiation (adipogenesis) marker genes (*PPARG* and *CEBPA*) were evaluated following the downregulation of *NSRP1* expression. (**J**) The cells were subjected to adipogenic differentiation for 10 days downregulating *NSRP1* expression, and the assessment of lipid droplet formation was conducted using Oil Red O staining. Scale bar, 200 µm. * represents the *p*-value is less than 0.05, ** represents the *p*-value is less than 0.01, *** means the *p*-value is less than 0.001.

To evaluate the influence of NSRP1 on pre-adipocyte proliferation and differentiation, we conducted interference experiments by transfecting three siRNAs targeting *NSRP1*. The results indicated that si2 and si3 exhibited the highest interference efficiency during both proliferation and differentiation stages (Figure 7A). Consequently, *NSRP1* knockdown was achieved through transfection with si2 and si3 (Figure 7B). Subsequently, CCK-8 assays were performed to assess the rate of cell proliferation. Over time, the proliferation rate accelerated following si-NSRP1 treatment, as demonstrated by the results of the CCK-8 assay (Figure 7C). RT-qPCR and Western blot analyses confirmed an increase in the expression of proliferation marker genes, *Ki67* and *PCNA*, during pre-adipocyte proliferation post si-NSRP1 treatment (Figure 7D,E). EdU assay results exhibited a parallel trend, indicating an increase in the percentage of EdU-positive cells post si-NSRP1 treatment (Figure 7F,G). Moreover, RT-qPCR analysis validated a reduction in the expression of differentiation marker genes, *PPARG* and *CEBPA*, during pre-adipocyte differentiation following si-NSRP1 treatment (Figure 7H). Western blot analysis further substantiated after the successful interference of *NSRP1*, demonstrating a decrease in the expression of differentiation marker genes *PPARG* and *CEBPA* (Figure 7I). Oil Red O staining revealed a significant reduction in lipid droplet formation following si-NSRP1 treatment (Figure 7J). In summary, following the downregulation of *NSRP1* expression, there was an observed acceleration in the proliferation rate of pre-adipocytes, coupled with a diminished capacity for lipid droplet formation. This implies that the *NSRP1* has the capability to inhibit the proliferation of pre-adipocytes, and additionally, it plays a role in promoting lipid droplet formation during the adipogenic stage.

### 2.9. NSRP1 Promotes Adipogenesis by Regulating AS and Expression of CCDC18

**Figure 8:**
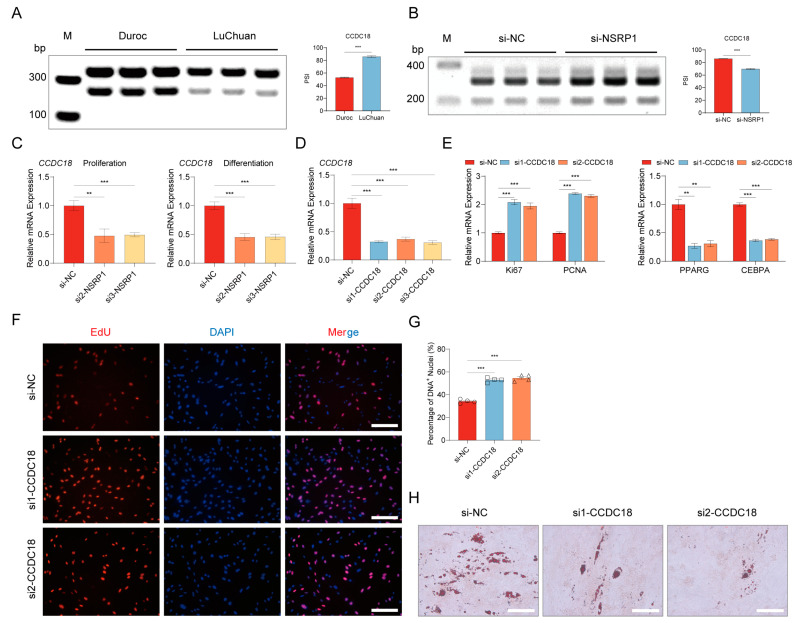
NSRP1 modulates adipogenesis through the regulation of AS and expression of *CCDC18.* (**A**) Semiquantitative RT-PCR analyses were conducted to assess splicing changes of *CCDC18* in representative Western pig breed (Duroc) and Eastern pig breed (Luchuan) adipose tissues. (**B**) Semiquantitative RT-PCR analyses were performed to evaluate splicing changes of *CCDC18* following downregulation of *NSRP1* expression. Left panel, the agarose gel electrophoresis results. Right panel, the quantification of alternative splicing levels through ImageJ (1.52i) processing of grayscale values. (**C**) The RT-qPCR experiment verified the effect of downregulating *NSRP1* on *CCDC18*. Left panel, proliferation stage. Right panel, differentiation stage. (**D**) The RT-qPCR results demonstrate the interference efficiency of three distinct small interfering RNAs targeting *CCDC18*. (**E**) The mRNA expression levels of cell proliferation and differentiation marker genes after si-CDDC18. (**F**) EdU assay was carried out after transfection si-CCDC18 for 24 h. Cells undergoing DNA replication were stained by EdU (red) and cell nuclei were stained with DAPI (blue). Scale bar 200 µm. (**G**) The percentage of DNA+ nuclei in (**F**) was quantified using ImageJ (1.52i). (**H**) The cells were subjected to adipogenic differentiation for 10 days downregulating *NSRP1* expression, and the assessment of lipid droplet formation was conducted using Oil Red O staining. Scale bar, 200 µm. ** represents the *p*-value is less than 0.01, *** means the *p*-value is less than 0.001.

*CCDC18* was a coDASG_DEG that was significantly highly expressed in Eastern pigs, which was found to be highly positively correlated with NSRP1 (*r* = 0.96, *p* < 1.00 × 10^−16^). An SE-type alternative splicing event occurred at exon 23 (100 nucleotides) of the *CCDC18* gene. To validate differential alternative splicing events in *CCDC18* identified through transcriptome data analysis between Eastern and Western pig breeds, we extracted adipose tissues from the representative Western breed, Duroc; and the Eastern breed, Luchuan pig. Semiquantitative RT-qPCR experiments were conducted, revealing significant differences in the alternative splicing events of *CCDC18* between Eastern and Western pig breeds. Notably, the Percent Spliced In (PSI) values in Luchuan pig were markedly higher than those in Duroc (Figure 8A). Furthermore, we validated whether NSRP1, as the alternative splicing factor, could regulate the occurrence of alternative splicing in *CCDC18*. Semiquantitative results demonstrated a significant decrease in the PSI values of *CCDC18* when *NSRP1* was downregulated in pre-adipocytes (Figure 8B). This suggested that NSRP1 indeed has the capability to regulate the strength of alternative splicing events in *CCDC18*. Afterward, we further verified whether NSRP1 could regulate the expression of *CCDC18*. The RT-qPCR results revealed significant inhibition in the mRNA of *CCDC18* during both cell proliferation and differentiation stages following si-NSRP1 treatment (Figure 8C). The above results indicate that NSRP1 serves as the alternative splicing regulatory factor, not only modulating the occurrence of alternative splicing in *CCDC18* but also concurrently regulating the expression of *CCDC18*.

Therefore, we further explored the regulatory role of *CCDC18* in pre-adipocyte proliferation and differentiation. We synthesized three siRNAs targeting *CCDC18* and individually transfected them into pre-adipocytes. The interference efficiency of siRNAs was detected by RT-qPCR, and the results showed that the expression of *CCDC18* was significantly inhibited by all three siRNAs (Figure 8D). Among these siRNAs, si1 and si2 were selected to verify the function of *CCDC18* in cell proliferation and differentiation. RT-qPCR showed that the expression of proliferative marker genes (*Ki67* and *PCNA*) increased, and the expression of differentiation marker genes (*PPARG* and *CEBPA*) decreased (Figure 8E). The EdU assay results showed an increase in the percentage of EdU-positive cells (Figure 8F,G). Oil red O staining revealed a significantly reduced in lipid droplet formation (Figure 8H). In conclusion, *CCDC18* inhibits pre-adipocyte proliferation and promotes differentiation, which is consistent with the function of *NSRP1*.

## 3. Discussion

As a classical type of gene expression regulatory mechanism, AS plays a crucial role in trait formation [9,23,24,25]. However, there have been few studies on the regulation of phenotypic differences between Eastern and Western pigs by gene alternative splicing. In this study, we utilized transcriptomic data from multiple tissues of Eastern and Western pigs, combined with information on TFs, selection signals, SFs, and QTLs, to comprehensively analyze the differences in AS and its regulatory mechanisms in various tissues of Eastern and Western pigs. Furthermore, we identified marker genes that influence traits such as pig adipose deposition and meat quality.

### 3.1. ASEs Identification and Gene Functional Enrichment Analysis

The study found that the most abundant types of ASEs in all tissues were MEX and SE, consistent with other studies’ findings [26,27]. The common ASGs in the eight tissues accounted for 63.21–76.13% of each tissue, indicating a certain degree of conservation in the genes undergoing ASEs across tissues. Additionally, we observed tissue-specificity in both DASGs and DEGs, which is in line with previous studies [28,29,30]. Among them, adipose and blood tissues showed the highest tissue-specificity in DASGs and DEGs, and these two tissues exhibited the highest average number of ASEs per gene. There are many immune cells in the blood, which are related to the disease resistance of pigs. Disease resistance and fat content are two traits that differ greatly between Eastern and Western pigs [6,7,8]. The above results indicated that AS might ultimately lead to the differences between the two traits of Eastern and Western pigs through its effects on these two tissues.

The coDASG_DEGs in adipose tissue were mainly enriched in pathways related to adipose deposition, immune inflammation, and insulin resistance. Previous researchers have shown that pathways such as the Ras signaling pathway, FoxO signaling pathway, PI3K-Akt signaling pathway, insulin resistance, MAPK signaling pathway, and AMPK signaling pathway play essential regulatory roles in adipose deposition [31,32,33,34,35]. Inflammation response and the immune system are closely related to adipose deposition, as they can influence the function, metabolism, and differentiation of adipocytes, thereby affecting adipose deposition [36,37,38]. Studies have demonstrated that inflammation response and insulin resistance downregulation can promote adipose deposition [37,39]. In this study, the downregulation of immune inflammation-related pathways and insulin resistance pathways in Eastern pigs may also promote adipose deposition. These pathways and related genes may be vital in regulating adipose deposition in Eastern pigs. However, further research is needed to explore how these pathways and genes interact and jointly regulate pig adipose deposition.

### 3.2. Associations between TFs and AS

This study found that TFs played a significant regulatory role in gene AS. TFs are a class of proteins that regulate gene transcription by binding to specific sequences on DNA to initiate or suppress the transcription process [40,41]. They can also regulate splicing processes through interactions with splicing regulatory factors [15,16] or RNA-binding proteins [15]. This study revealed that approximately half of the coDASG_DEGs are significantly influenced by TFs, indicating the important regulatory role of TFs in gene AS. Notably, we identified a TF, ARID4A, in adipose tissue. This TF is under selection in Western pigs but shows high expression in Eastern pigs. Studies have shown that the ARID4A protein can form complexes with proteins such as SIN3A and HDAC1, participating in histone deacetylation modifications, regulating chromatin structure and accessibility, and thereby controlling gene expression and cellular functions [42,43]. ARID4A can influence cell proliferation and division processes by regulating the expression of cell cycle-related genes [44,45]. Moreover, *ARID4A* is considered a potential tumor suppressor gene [46,47]. However, research on the role of ARID4A in pig adipose deposition is limited.

In this study, we found a strong positive correlation (*r* > 0.9, *p* < 3.40 × 10^−14^) between *ARID4A* and genes such as *AHI1*, *AKAP9*, *ANKRD12*, *CCDC18*, *ENSSSCG00000037142*, *IFT74*, *KIAA2026*, *NEXN*, *PPIG*, *ROCK1*, *SMC4*, *TWISTNB*, *BAZ1A*, *EIF5B*, *NSRP1*, and *BBX*. Among these genes, *AHI1*, *AKAP9*, *ANKRD12*, *CCDC18*, *IFT74*, *NEXN*, etc., have been shown to play an important role in adipose deposition or obesity [48,49,50,51,52,53]. Moreover, *AHI1*, *AKAP9*, *ANKRD12*, *CCDC18*, *IFT74*, *KIAA2026*, *NEXN*, *PPIG*, *ROCK1*, *SMC4*, *TWISTNB*, *EIF5B*, and *NSRP1* were also identified as potentially related to adipose deposition in this study. ARID4A is also associated with many QTLs related to adipose deposition traits, suggesting that this transcription factor may have an important regulatory role in pig adipose deposition, which requires further investigation.

### 3.3. ASGs under Potential Selection

The phenotypic differences between Western and Eastern pigs are influenced by artificial selection during domestication [1,4,5]. In this study, we further identified 20 candidate SGs in both adipose and blood tissues, which intersect with coDASG_DEGs, indicating that these genes may have been driven by artificial selection during domestication and breeding. Specifically, in adipose tissue, we found that the *SOX5*, *ENSSSCG00000014414*, *PPP1R12A*, *ZNF148*, *TNFSF10*, and *ROCK1* genes were under selection in Eastern pigs and significantly upregulated in adipose tissue, suggesting their potential influence on adipose deposition. Moreover, previous research has shown that *SOX5*, *PPP1R12A*, *ZNF148*, *TNFSF10*, and *ROCK1* genes regulate adipose deposition processes [54,55,56,57,58,59]. In blood, the *MACF1*, *IL1RL1*, *MGA*, *TTC14*, *ANKRD17*, *PDLIM7*, *FGD3*, *NPRL3*, *ENSSSCG00000014060*, *ENSSSCG00000039214*, *CYB5A*, and *HAGH* genes were under selection in Eastern pigs, and the *MACF1*, *IL1RL1*, *MGA*, *TTC14*, and *ANKRD17* genes were significantly upregulated in Eastern pig blood. Previous reports have implicated these genes in affecting animal immunity and inflammation [60,61,62,63,64,65,66]. They might also play a crucial role in influencing the disease resistance of pigs. These findings suggested that the AS of these candidate SGs might have been subjected to artificial selection during pig domestication, and they could contribute to the phenotypic differences observed between Eastern and Western pigs in adipose deposition and disease resistance.

### 3.4. SF NSRP1 Promotes Adipose Deposition

SFs are a class of proteins that play a crucial role in the process of AS, acting as regulators and mediators [17]. This study found that the SF NSRP1 played an important role in promoting lipid droplet formation during the adipogenic stage. NSRP1 is a protein that regulates the formation and function of spliceosomes [67]. The spliceosome is a complex part of RNA splicing regulation, and its function is closely related to RNA splicing and post-transcriptional regulation [68]. Therefore, NSRP1 may influence the splicing regulation of genes related to lipid metabolism, thereby affecting adipose deposition. In this study, we discovered that the splicing factor NSRP1, along with 17 highly correlated genes (*r* > 0.86, *p* < 6.10 × 10^−12^), is located in QTLs associated with pig adipose deposition. Among them, *ANKRD12*, *ARID4A*, *IFT74*, *KIAA2026*, and *ROCK1* are located in multiple fat-related QTLs, and *ANKRD12*, *CCDC18*, *NEXN*, *PPIG*, and *ROCK1*, are present in the intramuscular fat content QTL. Reports have already shown that ANKRD12, NEXN, IFT74, and *ROCK1* play an important role in adipose deposition or obesity [50,52,53,56]. While *CCDC18* is a new marker gene for fatty liver in chicken [51], its role in fat deposition has been rarely reported. The present study showed that *CCDC18* was highly positively correlated with ARID4A (SG, TF, *r* = 0.94, *p* < 1.00 × 10^−16^) and NSRP1 (SF, *r* = 0.96, *p* < 1.00 × 10^−16^), implying that *CCDC18* might be regulated by transcription factor ARID4A and splicing factor NSRP1. Furthermore, this study verified for the first time through a series of experiments that NSRP1 promotes adipogenesis by regulating alternative splicing and expression of *CCDC18*. However, the role of the transcription factor ARID4A in this process needs further study.

### 3.5. Limitations of the Study

Adipose deposition in pigs is a complex process regulated by multiple factors, including genetics, nutrition, and metabolism [69,70,71]. Although this study has identified some genes and pathways that may be involved in adipose deposition, fully resolving this complexity remains challenging. Further research is needed to explore the interactions between these pathways and genes and how they work together to regulate adipose deposition in pigs. In experimental verification, it was found that NSRP1 promoted adipogenesis by regulating the alternative splicing and expression of *CCDC18*, and the transcription factor ARID4A might also play an important role in this process. However, further research is still needed to explore the function and mechanism of ARID4A in adipose deposition.

### 3.6. Commercial Prospects of Marker Genes

The marker genes identified in this study have an important impact and commercial value on pig meat quality improvement. By identifying key genes associated with adipose deposition, targeted genetic improvements can be achieved to improve adipose deposition levels and meat quality in pigs [72]. The discovery of these marker genes can not only help select pig breeds with moderate adipose deposition and excellent meat quality but can also speed up the breeding process and reduce breeding costs. In addition, the commercial application of these marker genes can also promote the development of the pork industry, meet the market demand for high-quality pork, and improve breeding efficiency and economic returns. However, to realize the commercial value of these marker genes, further in vivo or large-population validation of these genes is required.

## 4. Materials and Methods

### 4.1. Data Collection

We first selected the transcriptome data of Eastern (Est) and Western (Wst) pig breeds from the NCBI-SRA database to download. In the beginning, we downloaded the transcriptome data of 302 pigs. After quality control, we removed low-quality data and finally retained the transcriptome data of 243 individuals. The retained samples comprised eight tissues, such as adipose (37: Est = 11, Wst = 26), blood (59: Est = 10, Wst = 49), heart (18: Est = 9, Wst = 9), kidney (11: Est = 6, Wst = 5), lung (9: Est = 4, Wst = 5), muscle (55: Est = 34, Wst = 21), ovary (24: Est = 8, Wst = 16), and spleen (30: Est = 25, Wst = 5). For detailed information about the data, please refer to Table S2.

### 4.2. Transcriptome Data Quality Control and Alignment

Firstly, the collected sequencing data underwent quality control using the fastp (v0.20.0) software to eliminate adapter sequences and low-quality reads [73]. Subsequently, the fastqc (v0.11.8) software was employed to assess the quality of the clean data obtained after fastp quality control, ensuring that the data met the required quality standards before proceeding with the subsequent alignment [74]. Next, the Sscrofa 11.1 version of the reference genome and annotation files for pigs were downloaded from the Ensembl website (https://ftp.ensembl.org/pub/release-97/, accessed on 12 June 2020), and an index was constructed using STAR (v2.7.1) [75]. Finally, the STAR software (v2.7.1) was used to align the quality-assured clean data generated from the fastp quality control process.

### 4.3. Identification and Differential Analysis of Alternative Splicing Events

The output bam files from STAR were processed using rMATS (v4.1.0) to analyze alternative splicing events (ASEs) [76]. We investigated five types of AS, namely alternative 5′ splice site (A5SS), alternative 3′ splice site (A3SS), mutually exclusive exon (MXE), skipped exon (SE), and retained intron (RI). By applying a cutoff of FDR < 0.05 and |IncLevelDifference| > 5%, we detected differentially alternative splicing events (DASEs). Subsequently, we referred to the genes identified from differentially alternative splicing as Differentially Alternative Splicing Genes (DASGs).

### 4.4. Identification of Differentially Expressed Genes

First, we utilized featureCounts (v2.0.0) to compute gene expression levels and removed genes with an average count of less than one across all samples [77]. Subsequently, we employed the DESeq2 (v1.38.3) software to normalize gene expression levels and conducted further analysis to identify differentially expressed genes (DEGs) [78]. For this analysis, we applied the criteria |log_2_(fold change)| > 1 (|log_2_FC| > 1) and *p* < 0.05 to select DEGs.

### 4.5. Gene Functional Enrichment Analysis

We then identified the shared genes between DASGs and DEGs, referred to as coDASG_DEGs. These genes may be subject to AS regulation and display significant changes in expression. Subsequently, we conducted KEGG (Kyoto Encyclopedia of Genes and Genomes) pathway enrichment analysis on coDASG_DEGs using the R package clusterProfiler (v4.6.2) to explore their biological functions [79]. Pathways with *p* < 0.05 were considered significantly enriched for these genes.

### 4.6. Protein-Protein Interaction Network Analysis

We analyzed the Protein-Protein Interaction (PPI) of the coDASG_DEGs using the STRING database (https://string-db.org, accessed on 18 June 2021). Employing K-means clustering, we divided the coDASG_DEGs from each tissue into different clusters. Subsequently, we conducted functional enrichment analysis on genes within each cluster using clusterProfiler (v4.6.2) to study their biological significance.

### 4.7. Integrated Analysis of Transcription Factors, Selection Signals, Splicing Factors, and QTLs

We obtained a list of pig transcription factors (TFs) from AnimalTFDB v4.0 (http://bioinfo.life.hust.edu.cn/AnimalTFDB4/#/, accessed on 21 May 2022) and filtered for TFs among the DEGs in each tissue. Subsequently, we conducted a correlation analysis between the identified TFs and coDASG_DEGs to investigate their regulatory role in significant splicing differences between Eastern and Western pigs. We also retrieved the list of genes under selective sweep (SGs) from our previous publication for Eastern and Western pigs [6]. By intersecting this list with coDASG_DEGs, we studied potential coDASG_DEGs influenced by artificial selection during domestication. Splicing factors (SFs) play a regulatory role in the AS process; we further screened for SFs among the DEGs. Then, we performed a correlation analysis between these identified splicing factors and coDASG_DEGs to study their regulatory role in significant splicing differences between Eastern and Western pigs. Lastly, we downloaded pig QTLs information from PigQTLdb (https://www.animalgenome.org/cgi-bin/QTLdb/SS/index, accessed on 21 December 2022) and conducted functional analysis on the coDASG_DEGs highly correlated with SFs. All correlation analyses were performed using the R programming language through Pearson correlation analysis, and a *p*-value less than 0.05 was considered statistically significant.

### 4.8. Adipocyte Culture and Induced Differentiation

The stromal vascular fraction cells (SVF cells) from the Bama pig were generously provided by Dr. Yangli Pei at Foshan University. The cells were cultured in DMEM supplemented with 10% fetal bovine serum (FBS) and 1% penicillin/streptomycin (Gibco, Grand Island, NY, USA). Upon reaching full confluence and allowing fusion for a day or two, the growth medium was replaced with an induction medium to initiate cell differentiation. The cells were cultured until the sixth day, at which point the maintenance medium was changed, and the culture was continued until the lipid droplets reached maturity, with the fluid being refreshed every two days. The induction medium consisted of 1 mg/mL insulin, 0.5 mmol/L isobutyl-1-methylxanthine (IBMX), 1 mmol/L dexamethasone, 1 μmol/L rosiglitazone, and 10% FBS. The maintenance medium included 1 mg/mL insulin and 10% FBS. Transient transfections were carried out using Lipofectamine 3000 (Invitrogen, Carlsbad, CA, USA), following the manufacturer’s instructions. Additionally, on days 3, 6, and 9 of the adipogenic induction process, the cells were transfected with siRNAs. The nucleotide sequences information of the siRNAs used in the study is shown in Table S3.

### 4.9. RNA Extraction and RT-qPCR

Total RNA was extracted using Trizol (Invitrogen, Shanghai, China) following the manufacturer′s instructions. The quality and quantity of RNA were assessed using the NanoDrop 2000 (Thermo Fisher Scientific, Waltham, MA, USA). Quantitative real-time PCR (RT-qPCR) was conducted using Fast ChamQ Universal SYBR qPCR Master Mix (Vazyme, Nanjing, China) for mRNA on an ABI Step One Plus Real-Time PCR system (Applied Biosystems, Foster City, CA, USA). The 2^−ΔΔCt^ method was employed to analyze the relative expression levels of mRNA. β-actin was used as endogenous controls to normalize the expression of mRNA. The sequence information of primers used for RT-qPCR is shown in Table S4.

### 4.10. Western Blot Analysis

Proteins were extracted by RIPA buffer (Thermo Scientific, Waltham, MA, USA) supplemented with phosphorylase inhibitor (Roche 5892791001, Basel, Switzerland) and protease inhibitor (Roche 04693132001, Basel, Switzerland). The concentration of obtained protein was measured by the BCA kit (Beyotime, Shanghai, China). The proteins were separated in 10% sodium dodecyl sulfate-polyacrylamide gel electrophoresis (SDS-PAGE) gels (EpiZyme, Shanghai, China) and transferred onto 0.45 µm Hybridization Nitrocellulose Filter (NC) membrane (Merck, Rahway, NJ, USA), and then probed with antibodies following standard procedures. These membranes were blocked with 5% skim milk at room temperature for 2 h, and then incubated with primary antibodies at 4 °C overnight. Subsequently, the membranes were incubated with secondary antibodies at room temperature for 1 h. The following antibodies were used in the present work: NSRP1 (1:500, 21360-1-AP, Proteintech, Wuhan, China), GAPDH (1:50,000, 60004-1-Ig, Proteintech, Wuhan, China), β-actin (1:20,000, 66009-1-Ig, Proteintech, Wuhan, China), KI67 (ab16667; 1:1000; Abcam, Cambridge, UK), PCNA (1:5000, 10205-2-AP, Proteintech Group, Wuhan, China), PPARG (1:1000, 16643-1-AP, Proteintech Group, Wuhan, China), CEBPA (1:500, 18311-1-AP, Proteintech Group, Wuhan, China). Secondary antibodies: Goat Anti-Rabbit (ZB-2301, 1:1000, ZSGB-BIO, Beijing, China) and Goat Anti-Mouse (ZB-2305, 1:1000, ZSGB-BIO, Beijing, China).

### 4.11. Cell Counting Kit-8 Proliferation Assay

SVF cells were seeded into 96-well plates, and the proliferation of cells was assessed at 0 h, 24 h, 48 h and 72 h after transfection using the Cell Counting Kit-8 (CCK-8) (Beyotime C0038, Beijing, China). After incubation for 1 h, the absorbance at 450 nm was measured using a microplate reader.

### 4.12. 5-Ethynyl-2′-Deoxyuridine (EdU) Staining

Cells were seeded in 12-well plates and cultured until they reached 50% confluency. After that, they were transfected and allowed to incubate for 48 h. Following the 48-h transfection period, EdU staining was performed using the BeyoClickTM EdU Cell Proliferation Kit (Beyotime, Shanghai, China). Briefly, the cells were fixed in 4% paraformaldehyde and permeabilized with 0.5% Triton X-100. Next, the cells were stained with Click Additive Solution in the dark for 30 min, and the nuclei were counterstained with 4′,6-diamidino-2-phenylindole (DAPI) solution. Images were acquired using a Nikon ECLIPSE Ti microscope, and the ImageJ software (1.52i) was employed to calculate the proportion of EdU-positive cells.

### 4.13. Oil Red O Staining

After induction of adipogenesis, cells were washed three times with PBS (Gibco, Carlsbad, CA, USA) and then fixed for 30 min in 4% paraformaldehyde. The samples were rinsed twice with 60% isopropanol and dried for 30 min before being treated with 1 mL of the oil red O dye working solution. A microscope was used to observe the oil red O staining after adding 1 mL PBS (Gibco, Carlsbad, CA, USA) to the culture plate.

### 4.14. Semiquantitative RT-PCR Analysis of Alternative Splicing Events

PCR products were separated by 1.5% agarose gel in 1 × TAE buffer for 40 min at 120 V. Quantification of gels was performed by densitometry using ImageJ software (1.52i, National Institutes of Health) for analysis. The sequences of the primers (Sangon Biotech, Shanghai, China) are shown in Table S5.

### 4.15. Statistical Analysis

Alternative splicing events were automatically detected and quantified using the percent-spliced-in (PSI, C) metric based on long (L) and short (S) forms of splicing events presents (equation shown below). Briefly, a PSI value was given according to the ratio of the long form to the total form present (short form and long form) to characterize the inclusion of exon.

C = L/(L + S) (1)

The results are represented as the mean ± SD. Statistical analyses of the differences between groups were performed using Student’s *t*-test. Statistical significance was set at * *p* < 0.05, ** *p* < 0.01 and *** *p* < 0.001.

## 5. Conclusions

In the present study, we found that AS might play an important role in fat deposition and disease resistance in pigs, which might be affected by transcription factors, selection signals, and splicing factors. We identified the TF ARID4A and the SF NSRP1 in adipose tissue, along with genes such as *ANKRD12*, *IFT74*, *KIAA2026*, *CCDC18*, *NEXN*, *PPIG*, and *ROCK1* might play critical roles in adipose-deposition traits. For the first time, we further confirmed that NSRP1 regulates adipogenesis by regulating alternative splicing and expression of *CCDC18* through experimental verification. These findings contribute to a better understanding of the formation and regulatory mechanisms of meat quality traits. The identified marker genes can serve as molecular markers for pig genetic breeding and meat quality improvement practices, providing a scientific basis for enhancing and optimizing the pork industry.

## Figures and Tables

**Table 1:** Tissue-specific differentially alternative splicing genes and differentially expressed genes.

	Adipose	Blood	Heart	Kidney	Lung	Muscle	Ovary	Spleen
DASGs	4187	2898	173	242	315	244	584	1721
SDASGs	1740	909	41	63	80	56	101	280
SDASGs/DASGs	41.56%	31.37%	23.70%	26.03%	25.40%	22.95%	17.29%	16.27%
DEGs	840	960	178	218	358	310	1348	620
SDEGs	504	604	69	109	210	161	880	311
SDEGs/DEGs	60%	62.92%	38.76%	50%	58.66%	51.94%	65.28%	50.16%
coDASG_DEGs	83	164	0	4	4	2	15	65

DASGs = Differentially Alternative Splicing Genes; SDASGs = Tissue-specific DASGs; DEGs = Differentially Expressed Genes; SDEGs = Tissue-specific DEGs; DASGs: FDR < 0.05, |IncLevelDifference| > 5%; DEGs: *p* < 0.05, |Fold change| > 2; coDASG_DEGs: Common genes between DASGs and DEGs.

## Data Availability

The RNA-seq data was obtained from the NCBI-SRA database (https://www.ncbi.nlm.nih.gov/sra/?term=, accessed on 27 August 2019). The detailed information on the transcriptome data is shown in Table S2.

## Supplementary Materials

The following supporting information can be downloaded at: https://www.mdpi.com/article/10.3390/ijms25052874/s1.
